# Dr. John Archibald McDonnell

**Published:** 1913-11

**Authors:** 


Dr. John Archibald McDonnell, Buffalo, 1875, died at his home in Chicago, September 19, aged 66. He was Professor of Surgery in the Bennett Eclectic College and a member of the State Medical Society.

